# The Conservative Management of an Uncommon Case of Postpartum HELLP (Hemolysis, Elevated Liver Enzymes, and Low Platelet Count) Syndrome Complicated by Liver Hematoma Causing Gastric Outlet Obstruction: A Case Report

**DOI:** 10.7759/cureus.47951

**Published:** 2023-10-30

**Authors:** Fabio R Garrote, Miguel E Perez-Viloria, Charles Caltagirone, Carolina De La Cuesta

**Affiliations:** 1 Anesthesiology, Florida International University, Herbert Wertheim College of Medicine, Miami, USA; 2 Anesthesiology, Mount Sinai Medical Center, Miami Beach, USA; 3 Critical Care, Mount Sinai Medical Center, Miami Beach, USA

**Keywords:** ultrasound, embolization, multidisciplinary collaboration, conservative management, subcapsular hematoma, gastric outlet syndrome, hellp syndrome

## Abstract

Hypertensive disorders of pregnancy, particularly preeclampsia, are significant contributors to maternal and fetal mortality worldwide. HELLP (hemolysis, elevated liver enzymes, and low platelet count) syndrome constitutes a severe manifestation of preeclampsia. Subcapsular liver hematoma (SLH) is a rare complication of HELLP syndrome, resulting from blood accumulation between the liver parenchyma and the Glisson's capsule. We present a unique case of a pregnant patient with HELLP syndrome complicated by SLH, leading to gastric outlet obstruction (GOO). The patient's medical history, clinical presentation, diagnostic evaluation, and management are discussed. The patient, with a history of pregnancy-induced hypertension, presented with HELLP syndrome at 34 weeks of gestation. Elevated blood pressure, liver enzymes, and low platelet count were observed. Postpartum, the patient developed SLH causing GOO. Conservative management, including intravenous fluids, pain control, and a nasogastric tube, was employed. Imaging confirmed SLH and GOO. Multidisciplinary collaboration guided the treatment approach, emphasizing close monitoring, nonoperative strategies, and dietary adjustments. The patient's condition improved, and she was discharged on postpartum day 20.

This case report underscores the challenges of managing HELLP syndrome complications, especially SLH-induced GOO. Early diagnosis, appropriate medical interventions, and interdisciplinary coordination are pivotal in ensuring positive outcomes. Conservative management can be effective in stable patients, but timely recognition and monitoring remain crucial for averting potential complications. This case contributes to the limited literature on managing such complex scenarios and highlights the importance of tailored strategies in multifaceted medical conditions.

## Introduction

Hypertensive disorder of pregnancy or preeclampsia constitutes one of the leading causes of maternal and fetal mortality worldwide, resulting in complications in 2-8% of global pregnancies [1]. In preeclampsia, the clinical presentation of hemolysis, elevated liver enzymes, and low platelets (HELLP) syndrome presents as a severe, multisystemic disorder with an incidence of up to 0.9% in all pregnancies [2]. Both genetic and environmental factors have been associated with the etiology of this disorder; however, a history of HELLP pregnancy is a risk factor for developing this condition in future pregnancies [3]. Among the many complications associated with HELLP syndrome, the most severe are cerebral hemorrhage, acute respiratory failure, coagulation disorders, and premature abruption of the placenta. Moreover, hepatic complications such as rupture, hemorrhage, and hematomas have also been reported. One rare complication of this syndrome is subcapsular liver hematoma (SLH), which accounts for <2% of HELLP pregnancies and is associated with a maternal mortality rate of 17-59% [4].

SLH results from the accumulation of blood between the liver parenchyma and the capsule of Glisson [4]. While the pathogenesis of hepatic hematoma formation is complex and not well understood, it is thought to involve fibrin deposition, leading to platelet activation, thrombus formation, and occlusion of capillaries [5]. Severe blunt trauma is the most common etiology of SLH, but minor traumatic events can also lead to this pathology. Minhas et al. have reported a case of SLH secondary to using "faja" (fabric belt around the waist) in Hispanic females. Other causes include hemodialysis, liver biopsy, intra-tumor hemorrhage, and preeclampsia [6]. SLH progresses in two phases: (1) anatomical expansion with capsular distention and (2) rupture, leading to hemodynamic collapse [5,7]. Rupture of SLH is a feared complication that results in hemodynamic instability and requires urgent management involving transfusions and anti-hemorrhagic therapy [8].

Anatomical expansion of hematomas can result in various symptoms that can be missed in clinical practice. As a result of normal physiologic liver capsule distension that occurs during pregnancy, female patients in their second or third trimester of pregnancy presenting with pain, nausea, vomiting, anorexia, dyspnea, and pallor should raise concerns for a possible diagnosis of SLH [7]. Due to the anatomical proximity to the distal stomach, pyloric sphincter, and duodenum, a rare potential complication of SLH expansion is gastric outlet obstruction (GOO).

GOO is a clinical syndrome characterized by epigastric abdominal pain and postprandial vomiting due to mechanical obstruction. It results from any disease process that causes a mechanical impediment to gastric emptying. Mechanical causes and motility disorders are common culprits and are typically associated with abdominal pain, postprandial vomiting, early satiety, and weight loss [9]. This case report presents a unique and rare case of a pregnant patient who developed HELLP syndrome complicated by a subcapsular hematoma that led to GOO. We discuss the patient's medical history, clinical presentation, diagnostic evaluation, and management.

## Case presentation

The patient was a 37-year-old, gravida 3 para 1, presenting at 34 weeks and five days to the labor and delivery floor. Her past medical history was significant for pregnancy-induced hypertension, well-controlled gestational diabetes, and a short cervix requiring a cerclage during the current pregnancy. She was admitted to the labor and delivery floor for induction of labor due to presenting signs and symptoms of HELLP syndrome including systolic blood pressure readings of 160s associated with elevated liver enzymes (AST: 574 and ALT: 1004), and platelets of 42. The patient was started on magnesium sulfate for seizure prophylaxis along with betamethasone for fetal lung maturity. The patient delivered the following day via normal spontaneous vaginal delivery with a second-degree perineal laceration that was successfully repaired.

**Figure 1:**
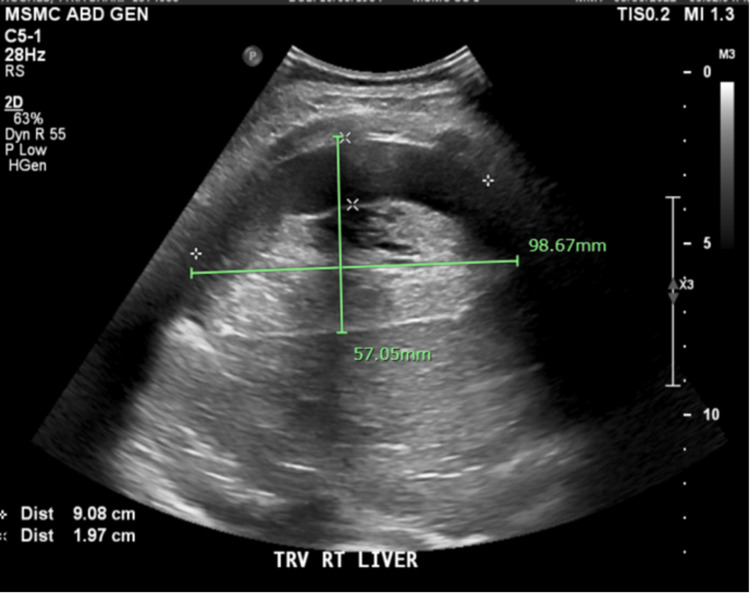
Right upper quadrant ultrasound

**Figure 2:**
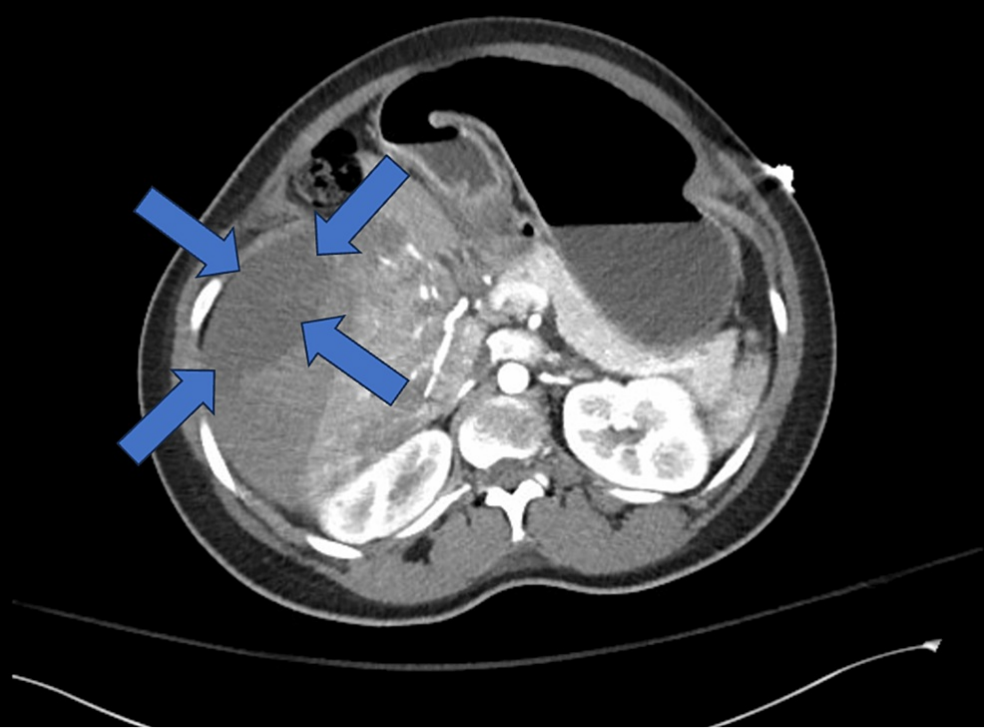
CT scan of abdomen and pelvis The blue arrows highlight the edges of the subcapsular hematoma CT: computed tomography

In the postpartum period, the patient experienced right upper quadrant pain, increasing abdominal distention, and decreased tolerance to oral intake. She became hypotensive and endorsed increased severity of right upper quadrant pain. During this time, the patient’s hemoglobin dropped from 12.9 g/dL pre-delivery to 10g/dL immediately postpartum. An ultrasound of the right upper quadrant showed fluid collection (Figure 1), and a CT abdomen and pelvis confirmed a large subcapsular hematoma. Radiological imagining showed active extravasation and mass effect on multiple organs, particularly the pylorus, causing GOO (Figure 2). A nasogastric tube was placed, the patient was transferred to the surgical ICU, and interventional radiology was consulted for the embolization of the suspected bleeding vessel. 

The patient was taken to the interventional radiology suite, and she underwent a successful embolization of two small vessels in liver segments 7 and 8 as well as bilateral embolization of uterine arteries. In the postoperative period, there was a decline in her hemoglobin levels to 6.4 d/dL and the patient was transfused with red blood cell products. A repeat CT abdomen and pelvis showed a right gonadal vein thrombosis and persistent hepatic post-embolization changes. 

On post-embolization day six, the patient’s abdominal pain started to resolve, and her liver function test (LFT) began to downtrend (AST/ALT: 266/888). At this time, the patient was not able to tolerate oral intake and was hence started on total parenteral nutrition (TPN). On post-procedure day nine, a CT abdomen and pelvis showed an interval decrease in the size of the right perihepatic hematoma. Her LFTs continued to downtrend (AST: 72 and ALT: 97) and her platelets normalized back to her pre-pregnancy value (238 x 10^3^/uL). A final CT abdomen and pelvis showed a decrease in the size of the subcapsular hematoma with no mass effect on surrounding organs. TPN was discontinued once the patient was able to consume an appropriate caloric intake. On post-embolization day 20, her diet was advanced to solid food without issues and two days later, the patient was discharged from the hospital.

## Discussion

Severe preeclampsia and HELLP syndrome can be managed by following three major options depending on gestational age. First, at 34 weeks of gestation: immediate delivery. Second, at 27 to 34 weeks: delivery within 48 hours, following maternal stabilization and corticosteroid treatment. Third, less than 27 weeks gestation: expectant management, and corticosteroid treatment with variable regimens. Additionally, it is recommended to terminate pregnancy if symptoms present before 24 weeks [10]. From a practical standpoint, approaching patients with diagnosed HELLP syndrome starts with evaluating the maternal status, gestational age, signs of active labor, and Bishop score. Necessary next steps include blood pressure monitoring, ultrasound examination, and lab tests, including complete blood count, AST/ALT, and coagulation parameters [10]. Management involves administering intravenous fluids, antihypertensive medication, and magnesium sulfate. To promptly reduce the risk of stroke, intravenous labetalol should be provided as a form of antihypertensive medication. Magnesium sulfate acts as a central nervous system depressant, effectively preventing convulsions and reducing the risk of recurrent seizures and maternal death [11]. Finally, corticosteroid therapy is frequently used to manage HELLP syndrome. Postpartum steroid therapy has been associated with significant benefits, including decreased hospital stay and a faster increase in platelet count, requiring fewer blood transfusions [12].

SLH is a rare complication of severe preeclampsia, and treatment should be tailored clinically depending on the patient. General recommendations include antibiotic therapy to reduce the risk of infections. Additionally, most patients presenting with SLH are hemodynamically stable since the Glisson's capsule maintains the hematoma, thereby decreasing blood loss. Thus, these patients are managed conservatively with intravenous fluids, transfusion of blood derivatives, and frequent monitoring. In some cases, percutaneous drainage of the hematoma might be required to achieve complete resolution. In hemodynamically unstable patients, embolization of bleeding arteries or surgical intervention is the main form of treatment. In general, conservative management (39.3%) is followed by surgical intervention (27.9%), percutaneous drainage (22.95%), and embolization (8.2%) [13].

**Figure 3:**
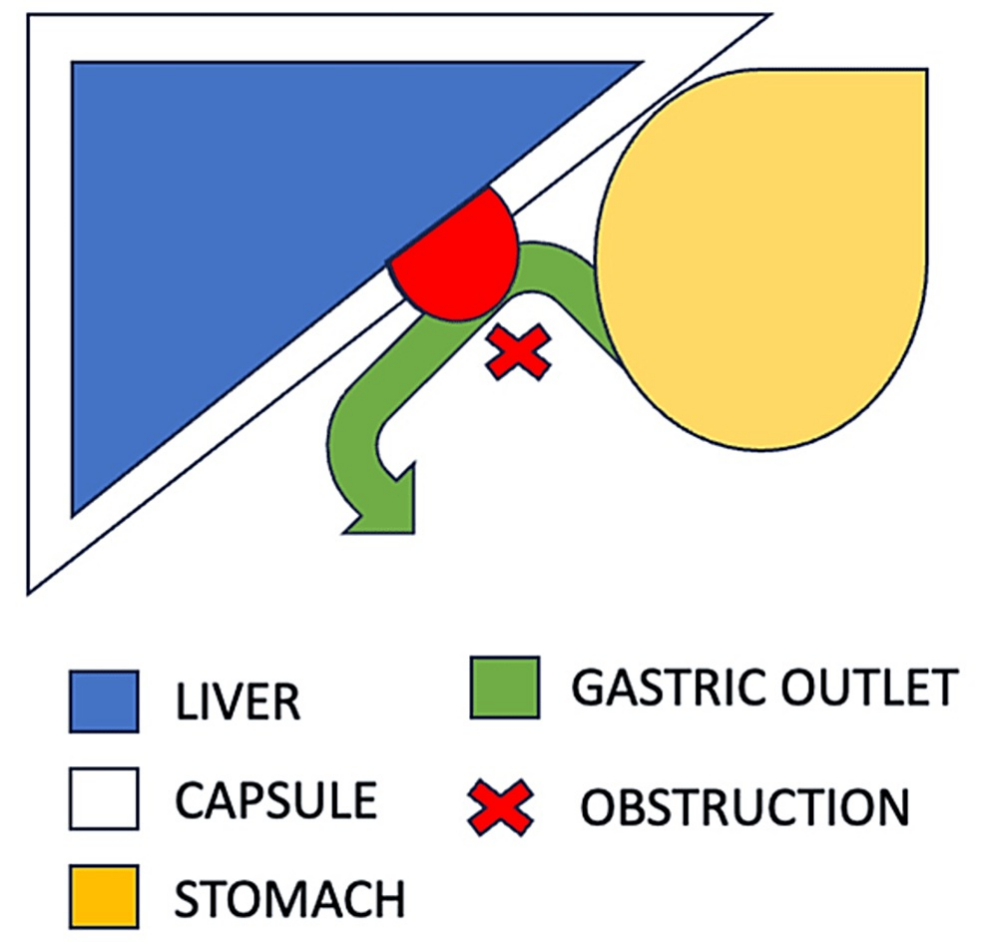
Diagram of anatomical concept

On the other hand, a rare complication resulting from SLH is GOO, which develops in the immediate postpartum period, as in our patient (Figure 3). There is extensive data in the literature on endoscopic therapy for the management of GOO. In our patient, GOO resolved after she received treatment for the underlying SLH and with the placement of a nasogastric tube and the provision of an NPO diet [14].

Our patient's condition was evaluated by a multidisciplinary team of obstetricians, general surgeons, and interventional radiologists. Given the hemodynamic stability of the patient, conservative management was recommended. The nonoperative approach and a wait-and-watch strategy were implemented with daily imaging studies to monitor the progression of the hematoma. The patient was on a clear liquid diet with intravenous hydration and parenteral nutrition. A nasogastric tube was inserted for gastric decompression, and the patient was started on proton pump inhibitors and anti-emetics to control symptoms. Blood transfusions were administered to maintain a hemoglobin level above 8 g/dL. Serial ultrasounds were obtained to monitor the hematoma size and the patient's clinical status. The patient's hemoglobin levels stabilized, and her pain and nausea improved by hospital day three with conservative management. A follow-up CT scan performed four days after the initial imaging showed no significant increase in hematoma size. The patient's diet was advanced to a low-fat, low-residue diet with a gradual transition to regular oral intake. The nasogastric tube was removed, and the patient was discharged on postpartum day nine.

**Figure 4:**
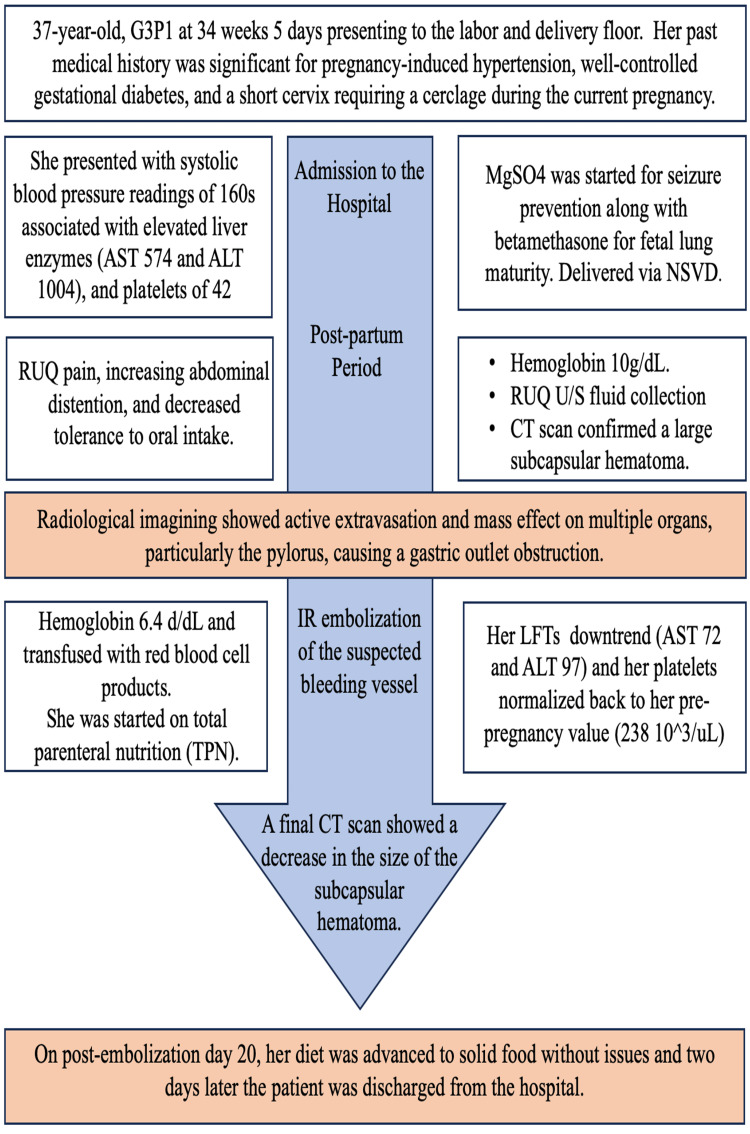
Timeline highlighting the case events

A timeline highlighting the case events is shown in Figure 4.

## Conclusions

This case report aimed to provide insights into the management of HELLP syndrome complicated by a subcapsular hematoma and GOO, a situation that is poorly described in the medical literature. HELLP syndrome is a severe, multisystemic disorder associated with significant maternal and fetal morbidity and mortality. The incidence of this disorder is relatively rare, but it can result in a wide variety of complications, including hepatic hematoma formation. SLH is a rare complication of HELLP syndrome and can result in significant maternal morbidity and mortality. The diagnosis of SLH can be challenging, as the symptoms can be nonspecific and can mimic other clinical conditions. Once there is a diagnosis or clinical suspicion of HELLP syndrome, management starts with an evaluation of maternal status, and gestational age. Lab studies, monitoring of vital signs, and ultrasonography constitute important next steps. To secure better health outcomes, the management involves providing the patient with intravenous fluids, antihypertensive medication, and magnesium sulfate. There is a significant association between postpartum corticosteroid therapy and reduced hospital stay and requiring fewer blood products. If the case is complicated by SLH, conservative management is recommended for most patients. Percutaneous drainage, embolization, or surgical intervention should be considered if the patient is hemodynamically unstable.

Our patient presented with GOO due to a subcapsular hematoma, which is an uncommon presentation of this disorder. The patient was successfully managed with strict bed rest, an NPO diet, and pain control. Her condition gradually improved over the course of her hospitalization and she was eventually discharged home without any significant complications. In conclusion, conservative management can be an effective treatment option for patients with HELLP syndrome complicated by SLH leading to GOO. However, early recognition of the diagnosis and close monitoring of the patient's clinical condition are essential to avoid potential complications.
